# The New Era of Pneumococcal Vaccination in Adults: What Is Next?

**DOI:** 10.3390/vaccines13050498

**Published:** 2025-05-07

**Authors:** Lale Ozisik

**Affiliations:** 1 Department of Internal Medicine, Faculty of Medicine, Hacettepe University, Ankara 06100, Türkiye; lale.ozisik@hacettepe.edu.tr; 2 Department of Vaccinology, Vaccine Institute, Hacettepe University, Ankara 06100, Türkiye

**Keywords:** *Streptococcus pneumoniae*, pneumococcal disease, pneumococcal vaccine, pneumococcal vaccine recommendations, adult vaccination, 15-valent pneumococcal conjugate vaccine, PCV15, 20-valent pneumococcal conjugate vaccine, PCV20, 21-valent pneumococcal conjugate vaccine, PCV21

## Abstract

*Streptococcus pneumoniae* remains the leading cause of community-acquired pneumonia in adults and bacterial meningitis in children worldwide. In addition to pneumonia, invasive pneumococcal diseases (IPDs), such as bacteremia and meningitis, pose a significant burden, particularly among older adults and individuals with underlying comorbidities. These diseases lead to substantial morbidity and mortality. Pneumococcal vaccination has been a cornerstone of disease prevention, reducing incidence and antimicrobial resistance. Recent advances in understanding *S. pneumoniae* epidemiology, genomic diversity, and the real-world impact of conjugate vaccines have driven the development and licensure of new-generation pneumococcal vaccines with expanded serotype coverage. Introducing 15-valent (PCV15), 20-valent (PCV20), and 21-valent (PCV21) conjugate vaccines has reshaped pneumococcal immunization strategies, particularly in adults, replacing previous sequential vaccine recommendations in many settings. In parallel, emerging epidemiological data and shifts in pneumococcal serotype distribution continue to influence vaccine policy decisions and immunization guidelines worldwide. In light of these advancements, adult pneumococcal vaccination recommendations continuously evolve to enhance protection in high-risk populations and optimize long-term immunity. This review provides an updated overview of the pneumococcal disease burden, the evolution of pneumococcal vaccines, and the latest immunization strategies in an expanding vaccine landscape. Additionally, we discuss future directions in pneumococcal vaccine development and the potential impact of novel vaccination approaches on public health outcomes.

## 1. Introduction

*Streptococcus pneumoniae* is the most common bacterial cause of community-acquired pneumonia in adults and a significant cause of bacterial meningitis in children worldwide. It was first was first isolated by Louis Pasteur in 1881. *S. pneumoniae* is a Gram-positive ɑ-hemolytic, facultative anaerobic microorganism that is human-specific. Most pneumococci are encapsulated with polysaccharides. Capsular polysaccharides (CPs) are major virulence factors and form the basis for classifying them by serotypes. More than one hundred serotypes were identified as of 2020, which are the target for current vaccines. Type-specific antibodies to capsular polysaccharides are protective against disease caused by that serotype. Some *S. pneumoniae* serotypes cause severe disease; other serotypes predominately cause asymptomatic colonization within the host nasopharynx. Asymptomatic nasopharyngeal carriage of *S. pneumoniae* is widely different across regions and is common, particularly in children. The prevalence rates range from 3 to 50% in healthy preschool-aged children, and lower rates of 5–10% reported in healthy adults [1,2]. Disease-specific serotype prevalence varies by age group and geographic area. A total of 20 to 25 predominant serotypes are responsible for nearly 90% of invasive pneumococcal disease cases [3].

Manifestations of *S. pneumoniae* range from asymptomatic nasopharyngeal colonization without immune clearance to milder diseases associated with the respiratory tract (otitis media, sinusitis, and pneumonia without bacteremia) or invasive diseases (meningitis, endocarditis, peritonitis, arthritis/osteomyelitis, and pneumonia with bacteremia). Pneumococcal pneumonia without bacteremia is the most common type of pneumococcal disease in adults. *S. pneumoniae* causes an estimated 10–30% of adult community-acquired pneumonia cases globally, and it is the most common bacterial cause of pneumonia that results in hospitalization [4]. Invasive pneumococcal disease (IPD) occurs in nearly 30% of hospitalized patients. On the other hand, the clinical burden is not restricted to IPD, but most often, the elderly experience cognitive declines, loss of independence, additional cardiac events, and finally, reduced lifespan following the infection, making it a global health concern [5].

### 1.1. Virulence

*S. pneumoniae* has a variety of virulence factors that enable it to colonize, invade, and cause disease. The most critical virulence factor is the polysaccharide capsule, which protects the bacterium from phagocytosis and complement-mediated immunity. Pneumolysin, a pore-forming toxin, contributes to host cell damage, inflammation, and immune evasion [6]. Surface proteins such as PspA and PspC. Autolysin (LytA), choline-binding proteins (e.g., CbpA), and Protein F mediate adherence and invasion of host tissues [7]. The bacterium also produces IgA protease, which degrades mucosal IgA and neuraminidase and cleaves sialic acids on host cells to enhance colonization. Pili provide nasopharyngeal adhesion, while sialic acid utilization and molecular mimicry help evade the host immune system. Together, these factors enable *S. pneumoniae* to transition from colonization to cause severe diseases such as pneumonia, meningitis, and bacteremia [8,9].

### 1.2. Antibiotic Resistance

From the dawn of the antibiotic era until the outbreaks in South Africa in the late 20th century, *S. pneumoniae* remained equally susceptible to all antibiotics active against this organism. Although *S. pneumoniae* was initially thought to develop resistance only against penicillin, over time, resistance has expanded beyond penicillin to include other β-lactams (e.g., cephalosporins, carbapenems), macrolides, lincosamides, tetracyclines, TMP-SMX, and fluoroquinolones. In the following decades, the resistance of *S. pneumoniae* to these agents had become a worldwide health problem. In 2017, the World Health Organization (WHO) included penicillin-non-susceptible *S. pneumoniae* as one of the 12 most critical pathogens that are a priority for new antibiotic development [10]. Antibiotic resistance rates vary widely depending on geographic regions and periods. Recent global surveillance data from the antimicrobial testing leadership and surveillance (ATLAS) program (2016–2021) reported declining ceftriaxone susceptibility rates in Asia (from 83% to 75%) and Latin America (from 94% to 86%), while Europe and North America maintained susceptibility rates > 95%. Asia recorded the highest ceftriaxone non-susceptibility, with China (33.9%) and South Korea (33.8%) leading, followed by Türkiye and India with rates between 10 and 20%. The overall susceptibility rates of *S. pneumoniae* isolates to penicillin, ceftriaxone, and ceftaroline were 63.4%, 94.0%, and 99.6% globally [11]. Penicillin resistance is driven by mutations in penicillin-binding proteins (PBPs), with penicillin-non-susceptible strains reaching over 80% in countries such as Tunisia and over 30% in Bulgaria, Brazil, and China. Macrolide resistance, mediated by *erm* and *mef* genes, has exceeded 90% in China and Japan and is high in Eastern Europe and Africa [8]. These geographic disparities in resistance and rising multidrug resistance emphasize the need for region-specific surveillance, specific treatment, and prevention strategies.

Both antibiotics and vaccines exert selective pressure that can drive genetic adaptations in *S. pneumoniae* through several mechanisms, including serotype switching via genetic recombination, expansion of non-encapsulated and non-vaccine serotypes that act as reservoirs for antimicrobial resistance genes, and proliferation of non-pneumococcal streptococci within shared ecological niches, which can facilitate horizontal gene transfer. These evolutionary strategies collectively enable the pathogen to circumvent therapeutic and preventive interventions, fundamentally reshaping its serotype distribution and antimicrobial resistance profile [12]. Several non-vaccine pneumococcal serotypes with notable antibiotic resistance have emerged in the post-vaccination era. Many of these emerging serotypes were linked to lineages exhibiting higher rates of antibiotic resistance, highlighting the challenges posed by serotype replacement in the context of vaccination programs [13]. After the introduction of PCV7, antibiotic-resistant non-PCV7 serotype, penicillin non-susceptible 19A strains have emerged and substantially began to decrease after being targeted in updated vaccine formulations (PCV13) [14]. Similarly, after the introduction of PCV13, non-PCV13 serotypes (11A, 15A, 22F, 23A, 33F, and 35B) that carry macrolide-resistance genes have shown increasing prevalence [15,16,17]. Serotypes 22F and 33F were incorporated into PCV15. Serotypes 11A and 15B, frequently linked to resistance, were incorporated into PCV20 [12]. Other resistant serotypes, such as 15A, 23A, 23B, and 35B, which have shown increasing prevalence in several regions and resistance to macrolides and β-lactams, were incorporated into PCV21 [15,16,17,18].

These findings highlight the urgent need to integrate genomic surveillance with vaccine policy and to consider serotype-independent strategies, such as whole-cell or protein-based vaccines, as part of a long-term resistance mitigation plan in the face of evolving antimicrobial resistance [12]. Therefore, adult vaccination strategies should be considered a component of broader antimicrobial stewardship and public health preparedness efforts. In this context, pneumococcal vaccination plays an increasingly important role not only in preventing disease but also in mitigating the spread of antibiotic-resistant strains.

## 2. Epidemiology and Burden

WHO estimated that 1.6 million deaths in 2005, including 1 million children, occurred due to *S. pneumoniae.* The introduction of pneumococcal conjugate vaccines (PCVs) in many low-income countries over the past decade, supported by the Global Alliance for Vaccines and Immunization (GAVI), has led to significant reductions in IPD and pneumococcal pneumonia, yet the global burden remains high. A systematic analysis of the Global Burden of Diseases Study 2016 estimated that *S. pneumoniae* was responsible for 1,189,937 deaths and 197·05 million episodes of lower respiratory tract infections in 2016 globally, making it the leading bacterial cause of adult community-acquired pneumonia [19]. In 2019, lower respiratory infections (LRIs) were the fourth leading cause of death globally and the leading cause of death in the African region [20]. According to the Global Burden of Disease Study 2019 estimations, although the distribution of LRI-related mortality varied by age group and pathogen, globally, *S. pneumoniae* accounted for 1,382,751 deaths, most of LRI deaths across all age groups [21]. Notably, the highest burden was observed among the elderly. Moreover, *S. pneumoniae* was the leading contributor to disability-adjusted life years (DALYs) attributable to LRIs worldwide, underscoring its significant impact on global respiratory health [22]. More recent analyses reflect improving trends but still substantial mortality: Despite declines in incidence during the COVID-19 pandemic, in another systematic analysis of lower respiratory infections, it was estimated that, in 2021, *S. pneumoniae* was responsible for 97.9 million episodes and 505,000 deaths globally. Most of these deaths occur in low- and middle-income countries, particularly in Africa and Asia, where access to vaccines and medical care is limited. *S pneumoniae.* was the most prevalent cause of community-acquired bacterial lower respiratory tract infections across different income-level settings [23]. Hospitalizations due to pneumococcal pneumonia are common, particularly among the elderly. A meta-analysis estimated that in 2015, about 6.8 million pneumonia cases in adults ≥65 required hospital admission worldwide, resulting in approximately 1.1 million in-hospital deaths in this older age group. Once hospitalized, case fatality rates are substantial; for instance, 30-day mortality in older adults hospitalized with pneumonia can approach 15–20% in many settings [24].

Before the COVID-19 pandemic, 2019 Centers for Disease Control and Prevention (CDC) estimates of IPD were 30,300 cases and 3250 deaths in the US. The incidence rate was 9 cases per 100,000 population [25]. Despite declines in incidence during the COVID-19 pandemic, 2022 CDC estimates of IPD were 27,770 cases and 3230 deaths in the US. The incidence rate was 8.3 per 100,000 population. Incidence rates rise with age, and among adults ≥65 years, IPD incidence was 17.2 per 100,000 population [26].

The burden of adult pneumococcal disease in Europe is also substantial. In the recent years, the notification rate of IPD in the EU has been 5 cases per 100,000 population. In 2022, EU surveillance reported ~17,700 confirmed IPD cases, with the highest incidence in adults aged ≥65 years (about 12.6 per 100,000). Among cases with known outcomes, 12.8% died. The case fatality rate was 17.1%, the highest among cases aged ≥65 years [27].

In low- and middle-income countries (LMICs), the burden of pneumococcal disease among adults remains substantial and is often underrecognized because of limited surveillance systems. According to the Global Burden of Disease Study 2021, lower respiratory infections, including those caused by *S. pneumoniae*, continue to be a leading cause of morbidity and mortality in LMICs, particularly in sub-Saharan Africa and South Asia [23]. Estimations from the same study showed that the highest rates of lower respiratory infection mortality caused by *S. pneumoniae* among all ages in 2021 were in Chad, South Sudan, Guinea, Somalia, and Central African Republic 2021. For instance, regions of sub-Saharan Africa with high HIV prevalence have extremely high IPD rates in adults. A surveillance study in Malawi (pre-PCV era) found an adult IPD incidence of 58 per 100,000 years, peaking at ~109 per 100,000 in 35–40 years (an age group with high HIV co-infection) [28]. Even after the implementation of PCV childhood vaccination programs, many LMICs still face a “double burden” of pneumonia deaths among both children and older adults because of wider health disparities. Limited access to healthcare, lower adult vaccination coverage, and risk factors such as HIV, malnutrition, and indoor air pollution in LMIC populations contribute to higher pneumococcal disease rates [23].

Risk factors for severe pneumococcal disease in adults are well recognized (Table 1). Older people and people with chronic medical conditions are at risk of IPD and pneumococcal pneumonia. The age-specific incidence of IPD in adults with comorbidities such as cardiovascular disease, diabetes mellitus, chronic lung disease, and alcohol abuse is higher than in healthy adults. The most frequently reported comorbid disease in patients with community-acquired pneumonia is chronic obstructive pulmonary disease (COPD); one in four hospitalized patients with pneumococcal pneumonia also has COPD [29]. Pneumococcal pneumonia is estimated to account for approximately 12–13% of all hospital-treated pneumonia cases in the US, contributing to nearly 225,000 adult hospitalizations each year [30]. The case fatality rate for all patients with IPD is approximately 10%, and it is much higher for the elderly (~35%) and patients with risk factors [31]. Given the dire issue of pneumococcal disease burden globally, vaccination remains a high priority.

While childhood pneumococcal mortality has declined, the high disease burden in adults, particularly in resource-limited countries, remains a critical concern [23]. Strengthening adult pneumonia surveillance and expanding prevention in LMICs is vital to reduce the substantial morbidity and mortality attributable to pneumococcal disease in adults.

## 3. Evolution of Pneumococcal Vaccines

Vaccines were first developed against the most burdensome serotypes at the beginning of the 20th century. The first vaccines used purified, bacterial-derived CPs to activate the immune system that could not activate the adaptive immune system, providing a low-affinity, very short-lived IgM response. The first pneumococcal polysaccharide vaccine was a 14-valent pneumococcal polysaccharide vaccine (PPSV14), licensed in the US in 1977. In 1983, PPSV14 was replaced by PPSV23, which included 23 serotypes associated with the majority of invasive pneumococcal disease cases. Using CPs alone as an antigen led to the secretion of IgM antibodies by a specific B cell clone that could recognize CPs via the B cell receptor. However, most pure CPs cannot trigger T cell help to induce antibody class switching, affinity maturation, and memory B and T cell production, which are important for effective and prolonged protection. The immunity conferred by PPSV23 would wane in 3–5 years without the activation of T cell-mediated immunity [32].

Although it was effective in adults and elderly populations, it was not recommended for infants who are at high risk for IPD because of their immature T-cell-independent immune response [33].

To overcome these challenges, in 2000, pneumococcal conjugate vaccines (PCVs) were developed based on the concept pioneered by Hib conjugate vaccines, which used carrier proteins such as diphtheria toxoid (PRP-D), meningococcal outer membrane protein (PRP-OMP), CRM_197_, and tetanus toxoid (PRP-T) [34]. The covalent conjugation of the CPs to an immunogenic carrier protein provided the induction of adaptive immune response via T cell by IgM to IgG antibody class switching, affinity maturation, and immune memory. Conjugation transformed the T cell-independent polysaccharide vaccines into T cell-dependent antigenic vaccines that were much more immunogenic in infants and adults [35,36,37].

**Figure 1:**
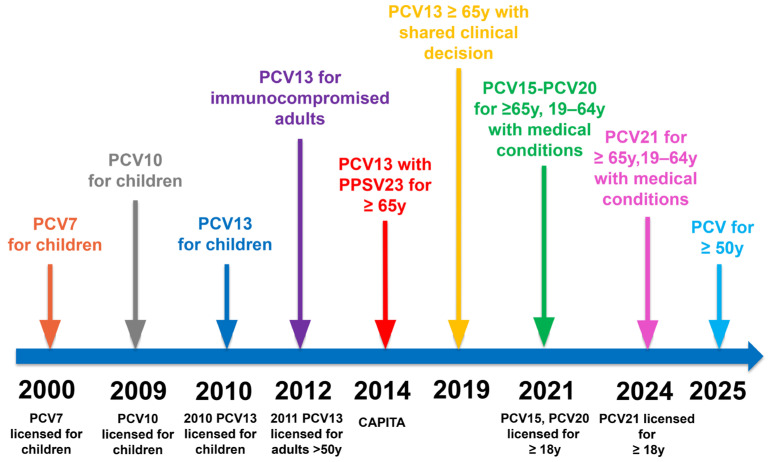
Evolution of Conjugated Pneumococcal Vaccines.

The introduction of PCV7 in 2000 for infants, which generated long-lasting immunity and reduced nasopharyngeal carriage for the seven most virulent serotypes of *S. pneumoniae*, has led to herd immunity. Following the introduction of PCV7, a substantial reduction in PCV7-covered type IPDs was observed, especially in children. In comparison, some indirect protection was noted in adults in countries with routine PCV7 pediatric immunization programs [38]. In 2009, PCV10 was developed and licensed for pediatric use [39]. Figure 1 illustrates the chronological evolution of pneumococcal conjugate vaccines, highlighting major milestones in licensure and recommendation changes across pediatric and adult populations.

In 2010, a 13-valent pneumococcal conjugate vaccine (PCV13) was approved for infants, covering additional serotypes [40]. In 2011, the United States Food and Drug Administration (FDA) approved PCV13 for adults 50 years of age and older based on immunogenicity studies [41]. Its clinical efficacy in adults 65 years of age and older was later confirmed by the landmark CAPITA trial, which demonstrated significant protection against vaccine-type community-acquired pneumonia and invasive pneumococcal disease [4]. These findings led to ACIP’s recommendation of PCV13 in adults ≥65 years in 2014, although later in 2019, policy adjustments introduced a more individualized approach in immunocompetent adults due to herd effects [42,43].

Studies showed that while childhood PCV10/PCV13 programs initially reduced invasive pneumococcal disease (IPD), incidence began rising again after 2015 because of non-PCV13 serotype replacement. By the year 2018, these non-vaccine serotypes caused a substantial proportion of IPD, especially in older adults. New expanded-valency formulations gradually replaced PCV13 [44].

In 2021, PCV15 and PCV20 were developed successively and licensed for adults, and in 2024, PCV21 was approved by the FDA [45,46,47].

**Figure 2:**
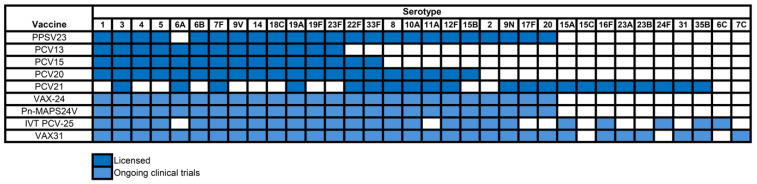
Serotypes of Conjugated Pneumococcal Vaccines.

The serotypes included in the pneumococcal vaccines are illustrated in Figure 2.

Although pneumococcal conjugate vaccines have significantly reduced vaccine-type IPD, their impact has been mitigated by the phenomenon of serotype replacement. Since current vaccines target specific serotypes and do not provide immunity against non-vaccine serotypes, non-vaccine types (NVTs) have emerged substantially after the introduction of these vaccines. PCVs reduce the nasopharyngeal colonization of vaccine-type serotypes (VTs), leading to NVT colonization and exerting selective pressure on VTs, allowing NVTs to emerge. PCVs may also cause Pneumococci to undergo genetic recombination and change their capsule to an NVT, which is called capsular switching. PCVs can also enhance the dominance of NVTs through the herd effect.

After the licensure of PCV7, in many populations, there has been an increase in pneumococcal disease caused by non-PCV7 serotypes, especially serotype19A [48]. A review of 118 studies from 33 European countries (2010–2022) found that serotypes covered only by PCV20 (e.g., 8, 10A, 11A, 12F, 15B, 22F, and 33F) have become increasingly prevalent in adults with invasive and non-invasive pneumococcal disease after the introduction of PCV13. These serotypes are associated with higher disease severity, mortality, and antimicrobial resistance and affect elderly and high-risk individuals more. Since 2018/2019, they account for ~60% of IPD cases in European adults [49]. Serotype replacement varies by region and population and remains a major driver behind the push for expanded valency and serotype-independent vaccine strategies [50].

Therefore, it is necessary to add new serotypes to vaccines. The continuous addition of serotypes results in large amounts of carrier protein. Because there would be competition for specific T cells that can recognize the protein components, increasing the number of serotypes may impair the antibody response to each serotype. The current cumulative approach of continuously adding serotypes to the formulation causes possible evolutionary dynamics to change and possible weakening of the immune response as the range of antigens given with each vaccine increases. The important immunological explanation for the observed reduction in serotype-specific immunogenicity as the valency of pneumococcal conjugate vaccines increases is carrier-induced epitope suppression (CIES). This phenomenon occurs when multiple polysaccharide antigens are conjugated to the same carrier protein or in individuals with pre-existing immunity to the carrier protein. Reduced immune responses to the polysaccharide components can result from one or more of several mechanisms [51,52,53,54]. First, competition for T-cell help may arise: polysaccharide-specific B cells depend on T-cell assistance for activation, but if carrier-specific B cells are more abundant or have higher affinity, they may dominate the interaction with T-helper cells, thereby impairing polysaccharide-specific antibody production. Second, in individuals with existing anti-carrier antibodies, these antibodies may bind to the carrier portion of the vaccine, masking the polysaccharide antigen and preventing its recognition by B cells and subsequent immune activation [55]. Third, repeated exposure to the same carrier protein can induce regulatory T-cell responses or other immunosuppressive pathways that attenuate the immune response to the conjugated polysaccharides [12].

Moreover, CPs must be modified before conjugation to activate carrier proteins for covalent binding when developing conjugated vaccines. This is achieved by oxidation of the cyclic diol structures in the CPs using sodium periodate. This process may alter the physicochemical characteristics of the CPs, leading to a product that does not allow MHC to present key antigens and making the conjugate vaccines less immunogenic [52]. It is evident that there is a need for developing new conjugation strategies and new vaccines that target the common structure of *S. pneumoniae,* which provide serotype-independent protection [52,56,57].

## 4. Recent Developments in Pneumococcal Vaccine Technology

### 4.1. Examples of Conjugate Vaccines with Novel Conjugation Strategies

**Pneumosil^®^**, a 10-valent pneumococcal conjugate vaccine developed by Serum Institute of India (Pune, India) in collaboration with PATH, was prequalified by WHO in 2020 and is currently used in pediatric immunization programs in Gavi-supported countries [58]. Designed by a different activation method reducing the alterations of physiochemical characteristics of CPs. CPs were conjugated through 1-cyano-4-dimethylaminopyridinium tetrafluoroborate chemistry to CRM_197_. Clinical trials have demonstrated its immunogenicity and safety, making it a viable alternative for national immunization programs, especially in Gavi-supported countries [59].

**Pn-MAPS24v** (GlaxoSmithKline, formerly ASP3772 [Astellas Pharma Inc., Northbrook, IL, United States]/AFX3772 [Affinivax, Inc., Cambridge, MA, USA]) is another novel 24-valent pneumococcal vaccine that uses a new multiple antigen-presenting system (MAPS) technology for CPs and carrier modification to overcome the limitations of traditional conjugate vaccines. This technology is based on the production of macromolecular complexes composed of biotin-modified CPs noncovalently attached to rhizavidin-fused carrier proteins that lead to the induction of robust B-cell and T-cell immunity in animal models. A phase 1/2 trial in adults was completed, and it was found to be highly immunogenic and well tolerated. The FDA granted it Breakthrough Therapy Designation. A phase 3 trial is expected to start in the near term. Subsequently, it was found safe for the pediatric population in a phase 1 trial and undergoing phase 2 studies [60].

**Vax-24** is a 24-valent conjugate vaccine that recently finished phase 1/2 trials in adults and is going into phase 3 trial. Vax-24 is developed using a novel approach of cell-free protein synthesis to produce a variant of cross-reactive material eCRM^®^ as the carrier protein, copper-free click chemistry, and site-specific conjugation [61]. Selected lysine residues of CRM_197_ were replaced with azide-containing non-native amino acids to construct the eCRM^®^ featuring site-specific conjugation capabilities. VAX-24 achieves high polysaccharide-to-protein ratios with reduced carrier-mediated immune interference by incorporating non-natural amino acids outside immunodominant regions. This approach minimizes carrier suppression and allows better immunogenicity [62]. A phase 2 trial is ongoing in healthy infants. The FDA granted it Breakthrough Therapy Designation.

**Vax-31** is a 31-valent conjugate vaccine that recently finished phase 1/2 trials in adults and demonstrated that the vaccine was well tolerated and elicited vigorous opsonophagocytic activity immune responses across all 31 evaluated serotypes. Vax-31 is developed by the same company using the same approach as Vax-24. It covers PCV20 serotypes and an additional 11 serotypes (2, 7C, 9N, 15A, 16F, 17F, 20B, 23A, 23B, 31, and 35B). The phase 1/2 study of VAX-31 The FDA granted Breakthrough Therapy Designation to VAX-31 for the prevention of IPD in adults. A phase 2 trial is ongoing in infants [63].

**IVT PCV-25** is a novel 25-valent pneumococcal conjugate vaccine under investigation in a phase 2 clinical trial in Canada, in adults [64]. It was developed using a new conjugation technology based on the bi-functional PEG linker, hydrazide-PEG-hydrazide, designed to create highly immunogenic and broad-spectrum conjugate vaccines [65].

The vaccine candidates described so far require a complex and costly manufacturing step of the conjugation process. To facilitate the conjugation process and significantly reduce the cost, a novel Protein Glycan Coupling Technology or bioconjugation is being investigated in the preclinical studies. This biological method uses genetically engineered bacteria (e.g., *Escherichia coli*) that can synthesize, link, and assemble the polysaccharide and protein components in a single organism rather than in separate chemical steps for conjugation. Preclinical studies support its potential for next-generation pneumococcal vaccines [66].

### 4.2. Emerging Pneumococcal Vaccine Technologies Beyond Conjugate Vaccines

To address serotype replacement, the continuous addition of serotypes to conjugate vaccines increased production costs while potentially reducing immunogenicity. As a solution, researchers have focused on developing whole-cell vaccines and protein/peptide-based vaccines, aiming to achieve serotype-independent protection. Whole-genome sequencing of clinical *S. pneumoniae* strains has also yielded valuable insights into bacterial characteristics and the extent of conservation among candidate antigens targeted for vaccine development.

#### 4.2.1. Protein-Based Vaccines

Protein-based vaccines represent a promising strategy for broadening protection against *S. pneumoniae,* particularly in adults, by overcoming the limitations of polysaccharide and conjugate vaccines. Unlike traditional formulations that rely on serotype-specific capsular polysaccharides, protein-based vaccines target conserved pneumococcal antigens, offering the potential for serotype-independent immunity with both humoral and T-cell responses.

For this purpose, multiple *S. pneumoniae* proteins that could be vaccine targets have been explored and employed in clinical trials. Various candidate pneumococcal protein antigens have been reported, including the pneumococcal surface proteins A (PspA) and C (PspC); choline-binding protein A (CbpA); pneumococcal choline-binding protein A (PcpA), structural protein; pneumolysin (Ply), a pore-forming toxin; lipoproteins (PlyLD); pneumococcal histidine triad protein D (PhtD), involved in metal uptake; PsaA, a manganese-binding adhesin; pneumococcal Serine-Rich Repeat Protein (PsrP) and sortase-dependent surface proteins [9,52]. Common target antigens and their functions in protein-based vaccines are listed in Table 2.

Various protein-based pneumococcal vaccine candidates have entered clinical development, and phase 1–2 clinical trials are ongoing [52,67,68].

**PnuBioVax** is a multi-antigen, serotype-independent vaccine that includes detoxified pneumolysin, PspA fragments, and pilus proteins. In a phase 1 study, it was well tolerated and elicited functional antibody responses in adults. A phase 2 trial is currently underway in the UK [69].

**A trivalent recombinant protein vaccine** composed of recombinant proteins PcpA, PlyD1, and pneumococcal histidine triad protein D (PhtD) has completed phase 1 trials in adults and infants. The vaccine was found safe and immunogenic, and clinical trials are ongoing [70].

Other candidates include fusion proteins such as **dPly–PhtD, monovalent PcpA subunit vaccines**, and peptide-fused formulations, most of which remain in preclinical or early clinical stages. Additionally, scientists are exploring viral-vectored protein vaccines—for example, an intranasal influenza virus encoding PspA has shown protection in mice—though these are in preclinical stages [71].

Despite significant progress and the development of several promising candidates, no protein-based pneumococcal vaccine has yet reached clinical licensure.

#### 4.2.2. Whole-Cell Vaccines

Whole-cell vaccines (WCVs) represent a promising serotype-independent strategy for pneumococcal disease prevention. WCVs use entire inactivated pneumococcal cells as the immunogen rather than specific antigens. They are developed by various methods such as attenuation, chemical treatment, heat, gamma irradiation, and preparation of whole-cell crude extracts to reduce or inactivate a pathogen’s virulence to maintain a serotype-independent humoral and cellular immunity. Among inactivation methods, gamma-irradiation preserved the structural integrity of the whole-cell vaccine most effectively, and the resulting Gamma-PN formulation was associated with robust antibody responses [72]. These vaccines expose multiple surface proteins, such as pneumolysin derivatives and pneumococcal surface proteins, to the immune system, inducing both humoral and cellular responses. Notably, WCVs have demonstrated the ability to stimulate T-helper 17 (Th17) cell-mediated immunity, which plays a crucial role in mucosal defense and clearance of nasopharyngeal colonization [73]. Preclinical studies have further underscored the potential of WCVs. In murine models, intranasal immunization with killed unencapsulated whole cells conferred protection against colonization and invasive disease caused by encapsulated pneumococcal strains [73]. Additionally, subcutaneous administration of a GMP-grade WCV demonstrated efficacy in preventing nasopharyngeal colonization and fatal aspiration sepsis in mice [74].

Phase 1/2 clinical trials of whole-cell vaccines, SPWCV + Alum, and PATH-wSP have been completed, and the results are promising. These studies reported that the vaccine was well-tolerated and elicited robust immunogenic responses, including increased IgG levels against pneumococcal proteins. They are all in the developmental stage [75].

The development of WCVs offers a cost-effective and broadly protective alternative to current pneumococcal vaccines, particularly beneficial for LMICs where diverse serotype prevalence and limited healthcare resources pose significant challenges. Ongoing research aims to optimize these vaccines’ formulations and assess their long-term efficacy and safety across different populations [76].

#### 4.2.3. mRNA and Nanoparticle Platforms

Currently, no pneumococcal mRNA vaccine has reached clinical trials, but the concept is actively being explored. Experts have noted that mRNA platforms (proven in COVID-19 vaccines) could be “readily adapted” for pneumococcal proteins. Preclinical studies are likely underway to evaluate immunogenicity in animal models, but specific candidates have not been publicly named. Ensuring the encoded antigen(s) effectively overcome the pneumococcus’s capsule shielding in vivo is a consideration. Also, mRNA vaccines may need optimization to induce mucosal immunity if the goal is to reduce colonization.

Given the geographic and temporal variability in serotype distribution and IPD incidence among adults, access to current, region-specific epidemiological data are critical for tailoring effective pneumococcal vaccination strategies. Therefore, continuous serotype-specific surveillance should be prioritized, particularly in countries where surveillance systems are still lacking [77]. In the era of next-generation pneumococcal vaccines, further clinical studies are warranted to evaluate long-term safety, durability of immune responses, and sustained protective efficacy in humans [4].

## 5. Effectiveness of Licensed Pneumococcal Vaccines

Recent data confirm that both PCV13 and PPSV23 are effective in preventing vaccine-type invasive pneumococcal disease (VT-IPD) and vaccine-type pneumococcal pneumonia (VT-PP) in adults. The CAPITA randomized controlled trial demonstrated PCV13 efficacy of 75% against VT-IPD and 45% against VT-PP in adults aged ≥65 years [4]. Observational studies supported these findings, with PCV13 effectiveness estimates ranging from 47 to 68% for VT-IPD and 38 to 68% for VT-PP. In contrast, PPSV23 showed moderate effectiveness, with pooled estimates of 45% against VT-IPD and 18% (95% CI: –4% to 35%) against VT-PP, though variability existed across settings and serotypes [56]. The difficulty in identifying vaccine-covered serotypes in pneumonia limits the interpretability of many earlier PPSV studies. Overall, both vaccines offer protection, but serotype-specific evaluation is essential for the accurate estimation of vaccine effectiveness. Further research is needed to evaluate the effectiveness of other previously licensed pneumococcal vaccines.

## 6. Cost-Effectiveness of Pneumococcal Vaccination

Pneumococcal vaccination has consistently demonstrated favorable cost-effectiveness in high-income and low- and middle-income countries (LMICs), particularly in high-risk adult populations such as the elderly and those with chronic comorbidities. Several health economic models have shown that pneumococcal conjugate vaccines (PCVs) are cost-saving or cost-effective in reducing the burden of invasive pneumococcal disease (IPD) and non-bacteremic pneumonia compared with no vaccination or polysaccharide vaccines alone. For example, PCV20 vaccination in adults aged ≥65 and those aged 18–64 years with underlying comorbidities in the UK is expected to prevent more hospitalizations, save more lives, and yield lower overall costs than current recommendations for PPV23 [78]. In LMICs, PCVs have demonstrated high cost-effectiveness through both direct protection and herd immunity, mainly where disease burden and antibiotic resistance are high [79]. The reduction in antibiotic use and prevention of antimicrobial resistance add further economic value to pneumococcal immunization programs. Despite the upfront cost, PCV use in adults is increasingly recognized as a high-value public health investment [80].

## 7. Pneumococcal Vaccination Coverage Rates in Adults

Pneumococcal vaccination coverage among adults varies significantly across regions. In the United States, the 2016 National Health Interview Survey data indicated that 66.9% of adults aged 65 years and older had received the pneumococcal vaccine. Among adults aged 19–64 years with increased risk for pneumococcal disease, coverage was 24.0% [81].

Pneumococcal vaccination coverage among European adults varies significantly across countries and age groups. A 2021 report highlighted that coverage rates for individuals aged 65 years and older range from 1% to 70%, with an average of 17.95% across 13 European countries [82].

Despite recommendations and reimbursement policies in Türkiye, adult pneumococcal vaccination rates remain very low. Limited studies report coverage rates of 2% to 9,8% for adults at risk, highlighting the need for increased awareness and implementation of vaccination programs [83,84].

## 8. Pneumococcal Vaccine Recommendations

### 8.1. United States

In the US, according to ACIP, pneumococcal vaccination is recommended for all adults aged ≥50 years and for adults aged 19–49 years with certain underlying medical conditions or risk factors. The recommended vaccines are PCV15, PCV20, and PCV21, and the schedule is determined by age, medical conditions, and prior vaccination history. ACIP pneumococcal vaccination recommendations for adults are listed in Table 3 [30].

In the US, before October 2024, ACIP recommended pneumococcal vaccination for children, all adults aged ≥ 65 years, and those aged 19–64 years with risk conditions for many years. In October 2024, ACIP recommended a single dose of PCV for all PCV-naïve adults aged ≥ 50 years. Recommendations for PCVs among adults aged 19–49 years with risk conditions and PCV13-vaccinated adults have not changed from previous recommendations [30]. In June 2024, ACIP recommended PCV21 as an option for adults who are recommended to receive PCV [47].

### 8.2. Europe

**Figure 3:**
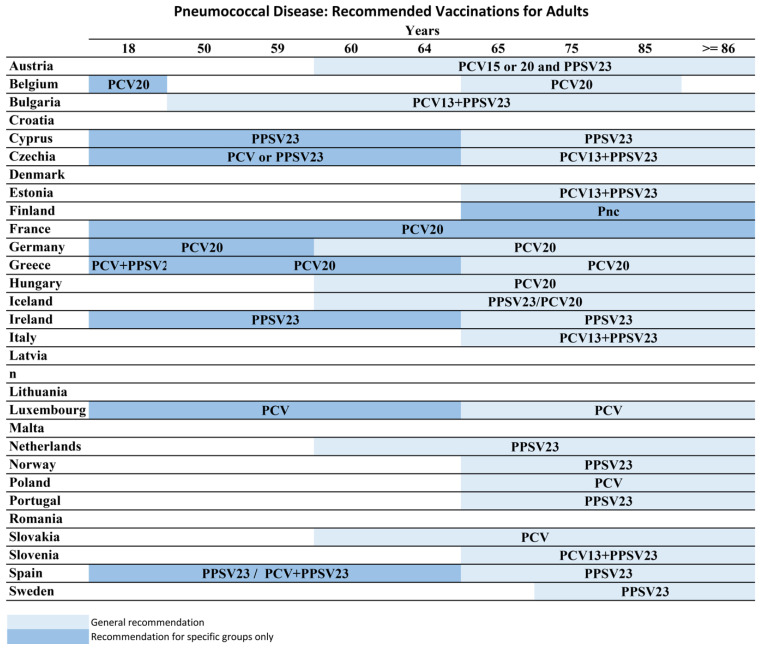
Pneumococcal Vaccine Recommendations in European Countries-ECDC, 2025.

In Europe, pneumococcal vaccine recommendations vary, with apparent discrepancies between Eastern and Western Europe. According to European Pneumococcal Vaccination: A Progress Report data, Pneumococcal vaccination is recommended for children in all countries across the EU/EEA except Estonia. 60% of countries recommend pneumococcal vaccination to children, clinical risk groups, and older people. Every country defines the risk groups and age criteria differently. Pneumococcal vaccine recommendations by age in European Countries are illustrated in Figure 3. Although they have different reimbursement strategies, many Europeans have to pay for pneumococcal vaccination. Only 15 countries reimburse or cover the cost for all three groups through their national healthcare systems [82]. That can be the reason for low coverage rates.

The contents of this report are covered by the ECDC legal notice. See: https://ecdc.europa.eu/en/legal-notice (accessed on 27 March 2025). The report reflects the state of submissions in the ECDC vaccination schedule platform as of 27 March 2025 [85].

### 8.3. WHO Regions

The literature has limited data on adult pneumococcal vaccine recommendations from countries in the WHO region other than North America and Europe. In a recent review analyzing WHO regions (outside North America and Europe), including the Americas, Africa, Eastern Mediterranean, South East Asia, and Western Pacific, it is reported that for 65 of 161 countries where adult pneumococcal vaccination information is available, only 63% (41/65) include adult pneumococcal vaccination in the national vaccination programs. Among the countries with recommendations, 58.5% targeted both older adults and at-risk groups, 9.8% focused exclusively on older adults, and 31.7% on risk groups only, of which nearly one-third were age-restricted. Age-based recommendations were more frequent in Southeast Asia, the Western Pacific, and the Americas, with age thresholds ranging from 50 to 70 years. Notably, most countries with recommendations included both PCV and PPSV23, typically in a sequential strategy. This study highlights the limited global adoption of adult pneumococcal vaccination and suggests that policies should be expanded and adapted to reflect national health priorities and demographic risk profiles [86].

### 8.4. Türkiye

In Türkiye, according to the adult vaccination guidelines, pneumococcal vaccination is recommended for adults aged ≥65 years and for those aged 19–64 years with risk conditions. With PCV15 and PCV20 becoming licensed and accessible in our country as of 2024, a significant simplification has been achieved in the adult pneumococcal vaccination algorithm. A single dose of PCV20 is recommended for individuals aged 65 and over and adults with chronic diseases. These individuals do not require additional PPSV23. In high-risk groups such as immunocompromised individuals, asplenic patients, those with CSF leaks, or those with cochlear implants, one of two alternative approaches can be applied: As the first option, starting with PCV15 or PCV13 and completing it with PPSV23 at least 8 weeks later; as the second option, vaccination with a single dose of PCV20 is possible. Healthcare professionals should decide this approach according to both individual risk levels and access to the vaccine [87].

## 9. Surveillance Gaps, Equity, and Ethical Considerations

### 9.1. Surveillance Gaps

Limited surveillance of IPD in LMICs remains a critical barrier to evidence-based vaccine policy. Without robust data on adult disease incidence and circulating serotypes, countries may struggle to prioritize adult vaccination or select the most appropriate vaccine formulation. According to the WHO’s Global Roadmap for Pneumococcal Vaccines in Adults (2022), only 33% of LMICs report national IPD data stratified by age and serotype; there is an inadequate evidence base related to the burden of disease [88]. The importance of extending immunization across the life course was first highlighted in the Global Vaccine Action Plan (GVAP) 2011–2020 [89] and later reaffirmed as a core strategic priority within the Immunization Agenda 2030 (IA2030) [90]. GAVI also emphasizes that poor disease surveillance leads to delays in vaccine introduction and underestimates disease burden in vulnerable populations [91]. Despite these global commitments, many low and LMICs still lack policies or national programs targeting adult and older adult populations for vaccination. Strengthening laboratory capacity, improving reporting systems, and investing in sentinel IPD surveillance, especially among older adults, is key to supporting effective, equity-driven immunization programs.

### 9.2. Equity and Ethics

Equitable access to pneumococcal vaccination is a central ethical obligation in adult immunization policy. The benefits of immunization are not spread equally, either among or within countries and in populations such as migrants, refugees, and internally displaced populations in fragile conflict-affected communities. According to recent data from the WHO, in 2023, the number of zero-dose children is 14.5 million, 60% of whom live in LMICs [92]. In many LMICs, adults with chronic illnesses, older persons, and those facing systemic barriers to care continue to be underserved. COVID-19 disruptions have also widened immunization inequities.

The ethical foundations of adult immunization policies are guided by global frameworks such as the WHO Strategic Advisory Group of Experts (SAGE) Values Framework, which emphasizes equity, fairness, and prioritization of marginalized and high-risk populations in vaccination strategies [93]. Historically, the GVAP, (2011–2020) endorsed by the 194 Member States of the World Health Assembly in 2012; afterward, the IA2030 endorsed in 2021, are frameworks for accelerating equitable vaccine coverage across the life course [89,90] GAVI, the Global Alliance for Vaccines and Immunization is a public-private partnership established in 2000 bringing together WHO, UN agencies, governments, donor countries and the private sector to expand equitable access to vaccines in LMICs [91]. IA2030, the global vision for vaccines and immunization for the decade ahead, explicitly recognizes immunization as a contributor to 14 of the 17 Sustainable Development Goals (SDGs), including poverty reduction, gender equity, education, and universal health coverage. One of the seven strategic priorities of IA2030 is coverage and equity [90]. It emphasizes the role of adult vaccination in promoting health equity, strengthening resilient health systems, and achieving broader social and economic goals, such as reducing poverty, advancing gender equality, and promoting inclusive societies. Gavi’s co-financing mechanisms similarly aim to reduce disparities by supporting sustainable access to adult vaccines in fragile health systems [91]. Integrating adult pneumococcal immunization into broader ethical and developmental agendas is essential for building resilient, inclusive health systems and ensuring vaccination strategies reflect global priorities such as universal health coverage, equity, and the SDGs. This integration defines adult pneumococcal vaccination not only as a medical intervention but as a pivotal instrument in advancing global health equity and social justice.

## 10. Conclusions

Despite remarkable progress in pneumococcal vaccine development, *S. pneumoniae* remains a significant cause of morbidity and mortality among adults worldwide. Advances in conjugate vaccine technology have enabled broader serotype coverage, and recent formulations mark a new era in adult pneumococcal immunization. However, the continuing emergence of non-vaccine serotypes, persistent antimicrobial resistance, and geographic variability in disease burden and vaccination uptake underscore the need for updated, region-specific strategies. Emerging technologies such as next-generation bioconjugation systems, recombinant protein antigens, and mRNA and nanoparticle platforms offer the potential for producing vaccines with broader, serotype-independent protection and more efficient manufacturing processes. Clinical development of whole-cell vaccines and T-cell–based immunogens may provide longer-lasting mucosal immunity and reduce nasopharyngeal carriage, further limiting transmission. Integration of these novel vaccines into immunization programs will require phase 3 trials, especially in adults with comorbidities and immunosuppression. Furthermore, real-world effectiveness data, genomic surveillance, and cost-effectiveness analyses will be critical to guide national and global vaccine policies.

In parallel, strengthening adult immunization infrastructure and integrating pneumococcal vaccination into broader public health initiatives by raising awareness and enhancing reimbursement strategies will be key to reducing the global disease burden.

## Figures and Tables

**Table 1:** Risk Factors for Invasive Pneumococcal Disease.

Age	Comorbid Conditions	Immunocompromising Conditions
>50 y	Chronic heart disease	Chronic renal failure, nephrotic syndrome
	Chronic liver disease	Immunodeficiencies
	Chronic lung disease	Iatrogenic immunosuppression
	Diabetes mellitus	Generalized malignancy
	Cochlear implant	HIV infection
	CSF leak	Hodgkin’s disease, leukemia, lymphoma, multiple myeloma
	Smoking	Solid organ transplant
	Alcoholism	Sickle cell disease or other hemoglobinopathies
	Nursing home residence	Congenital or acquired asplenia

**Table 2:** Common Target Antigens in Protein-Based Pneumococcal Vaccines.

Protein	Function	Rationale for Use
**Pneumolysin (Ply)**	Cytolytic toxin, pro-inflammatory	Highly conserved, induces strong neutralizing antibodies (used as toxoids in vaccines)
**PspA (Pneum surface prtA)**	Inhibits complement deposition	Surface-exposed, induces opsonic antibody, variable, families 1/2 cover ~99% of strains
**PhtD (Hist triad prt D)**	Zinc transport, adhesion	Surface-exposed, conserved; shown to be immunogenic and protective in animals
**PsaA**	Manganese-binding lipoprt (ABC transporter)	Adhesion, nutrient acquisition; conserved
**PcpA**	Cell wall surface protein (choline-binding)	Involved in colonization, induces mucosal and systemic immunity
**PspC/CbpA**	Complement-binding, adhesion to epithelial cells	Important in nasopharyngeal colonization
**Pili proteins (e.g., RrgB)**	Adhesion to host cells	Targeted in some vaccines (e.g., PnuBioVax)

**Table 3:** Pneumococcal Vaccination Recommendation for Adults, 2025, ACIP.

Age/Group	Recommended Vaccine	Interval
**≥50 years (no prior PCV)**	PCV15 + PPSV23/ PCV20/PCV21	PPSV23 ≥ 1 year after PCV15 (8 weeks if high risk)
**≥50 years (received PCV13)**	PCV20/PCV21	≥1 year after PCV13
**≥50 years (received PPSV23)**	PCV15/PCV20/PCV21	≥1 year after PPSV23
**≥50 years (received PCV13 + PPSV23, both before 65)**	PCV20/PCV21	≥5 years after last vaccine
**≥50 years (received PCV13 + PPSV23, after 65)**	PCV20/ PCV21 (Clinical decision)	≥5 years after last vaccine
**19–49 years with risk conditions (no prior PCV)**	PCV15 + PPSV23/ PCV20/PCV21	PPSV23 ≥ 1 year after PCV15 (8 weeks if high risk)
**19–49 years with risk conditions (received PCV13)**	PCV20/PCV21	≥1 year after PCV13
**19–49 years with risk conditions (received PPSV23)**	PCV15/PCV20/PCV21	≥1 year after PPSV23
**19–49 years with risk condition ^1^ (received PCV13 + PPSV23)**	PCV20/PCV21	≥5 years after last vaccine

^1^ For immunocompromising conditions, cochlear implant and CSF leak, a PCV is recommended. For alcoholism, chronic heart/liver/lung disease, cigarette smoking, or diabetes mellitus, no additional PCV or PPSV23 doses are recommended at this time. Review pneumococcal recommendations when age 50 years or older.
